# Ebstein Anomaly Associated With Primary Lymphedema: A Case Report and Literature Review of a Rare Anatomical Combination

**DOI:** 10.7759/cureus.117421

**Published:** 2026-10-04

**Authors:** Evangelos Dimakakos, Margarita G Toumanidou, Kyriaki Cholidou, Ioannis Papapanagiotou, Konstantinos Psarogiorgos, Stavros Toilos, Filothei Adonatou, Theodoros Mariolis-Sapsakos, Theodore Troupis

**Affiliations:** 1 Department of Nursing, School of Health and Care Sciences, University of West Attica, Athens, GRC; 2 3rd Department of Internal Medicine, Vascular Unit, Sotiria Thoracic Diseases Hospital of Athens, University of Athens, Athens, GRC; 3 Center of Prevention, Diagnosis and Treatment of Lymphedema - Lymphatic Diseases for Adults and Children, Metropolitan Hospital, Athens, GRC; 4 1st University Department of Respiratory Medicine, “Sotiria” Chest Diseases Hospital, Athens, GRC; 5 Department of Obstetrics and Gynecology, National and Kapodistrian University of Athens, Athens, GRC; 6 Department of Physiotherapy, Metropolitan General Hospital, Athens, GRC; 7 Department of Anatomy, Faculty of Medicine, School of Health Sciences, National and Kapodistrian University of Athens, Athens, GRC

**Keywords:** congenital heart disease, ebstein anomaly, lymphatic hypoplasia, lymphoscintigraphy, primary lymphedema

## Abstract

A 15-year-old male patient with a congenital heart disease known as Ebstein anomaly presented with edema of the right leg. The patient underwent lymphoscintigraphy, which revealed bilateral hypoplasia of the lower-extremity lymphatic system. The coexistence of these conditions may represent a previously unrecognized syndromic association. Physicians should be aware of the possible coexistence of these conditions and investigate patients with Ebstein anomaly in order to minimize physical and psychological complications.

## Introduction

Primary lymphedema can present either as an isolated condition or in association with congenital anomalies [1]. Ebstein anomaly is an uncommon congenital heart disorder characterized by tricuspid valve dysplasia and right ventricular abnormalities [2]. We present a case of a 15-year-old male patient with Ebstein anomaly and bilateral lymphatic hypoplasia diagnosed by lymphoscintigraphy, which represents a possibly unreported connection between congenital cardiac and lymphatic developmental anomalies.

## Case presentation

It is well known that sometimes primary lymphedema is associated with other congenital or acquired diseases [1]. In this study, we report a case of a 15-year-old patient who presented to the Vascular Unit of the 3rd Department of Internal Medicine at the Sotiria Thoracic Diseases Hospital of Athens, University of Athens, with severe right lower-extremity edema as the primary complaint. The swelling was first noticed eight months earlier, after air travel. No clinically apparent edema was observed in the left lower extremity, although red striae were present, despite abnormal lymphoscintigraphic findings. The patient had no family history of lower-extremity edema. However, the patient had a history of a congenital cardiac anomaly known as Ebstein anomaly, which is characterized by apical displacement of the septal and posterior tricuspid valve leaflets, leading to atrialization of the right ventricle with a variable degree of malformation and displacement of the anterior leaflet [2]. In 2024, the patient underwent replacement of the tricuspid valve due to severe heart failure. Postoperatively, the patient was followed up by cardiologists. He traveled 13 times by airplane between 2024 and the time of presentation, without experiencing similar symptoms during or following his previous flights. The right lower-extremity swelling was first noticed following his most recent air travel. Clinical examination revealed red striae on both legs and his body (he reported they had been present since 2022 and were presumed to be allergic in origin). The edema was firm and the thickness extended from the toes to the mid-calf of the right lower extremity. The skin overlying the mid-calf was soft, with mild edema extending to the knee (Figure 1).

**Figure 1:**
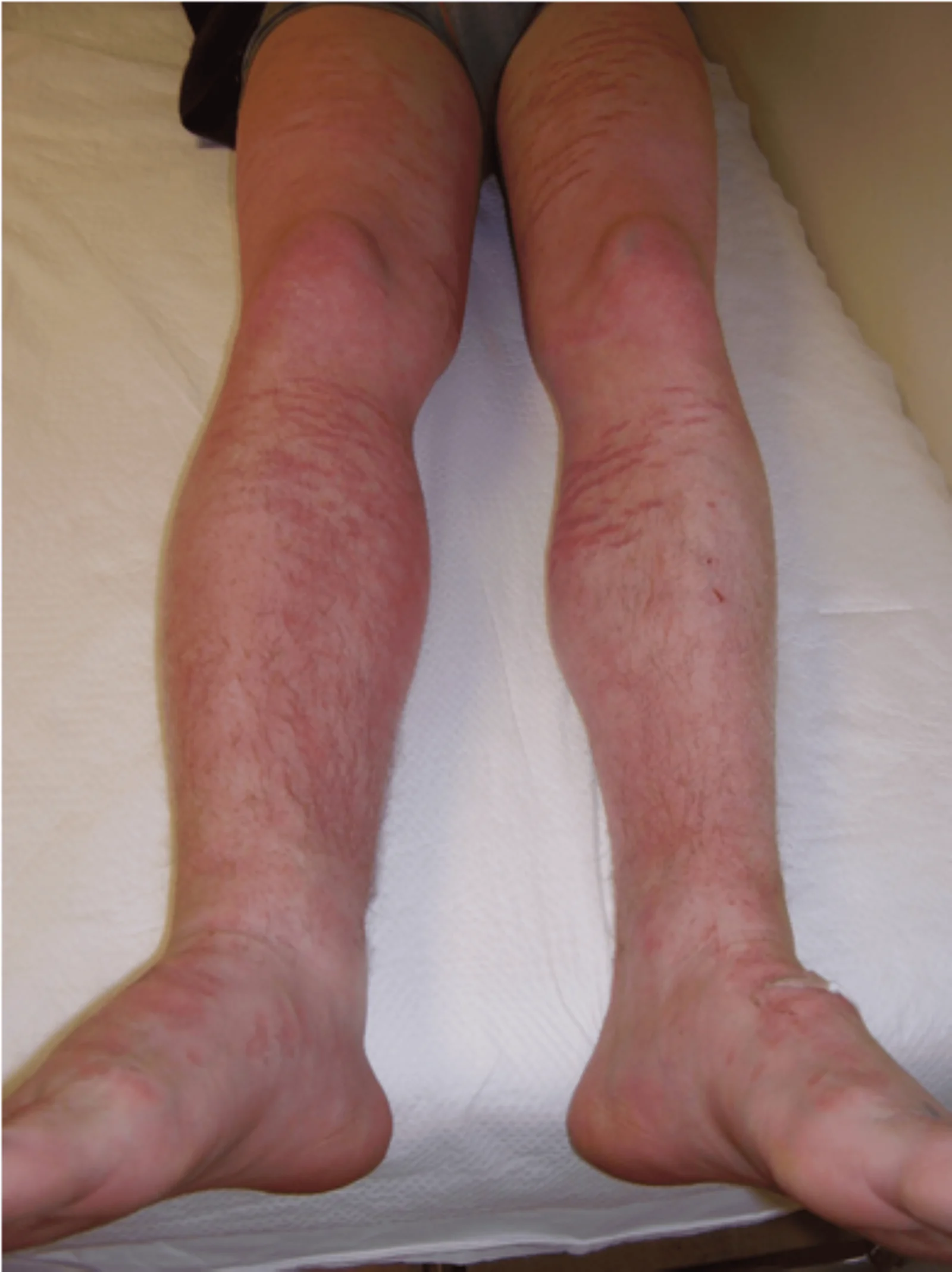
Severe right lower-extremity edema and red striae.

Stemmer’s sign was positive. Before presenting to our hospital, the patient underwent an abdominal CT scan and color duplex ultrasound of the veins of both lower extremities, as requested by his treating physician. The results of these examinations were reviewed during our clinical assessment and revealed no pathological findings in the abdominal organs or lower-extremity veins. Primary lymphedema was suspected. A subsequent lymphoscintigraphy revealed bilateral lower-extremity lymphatic hypoplasia (Figure 2).

**Figure 2:**
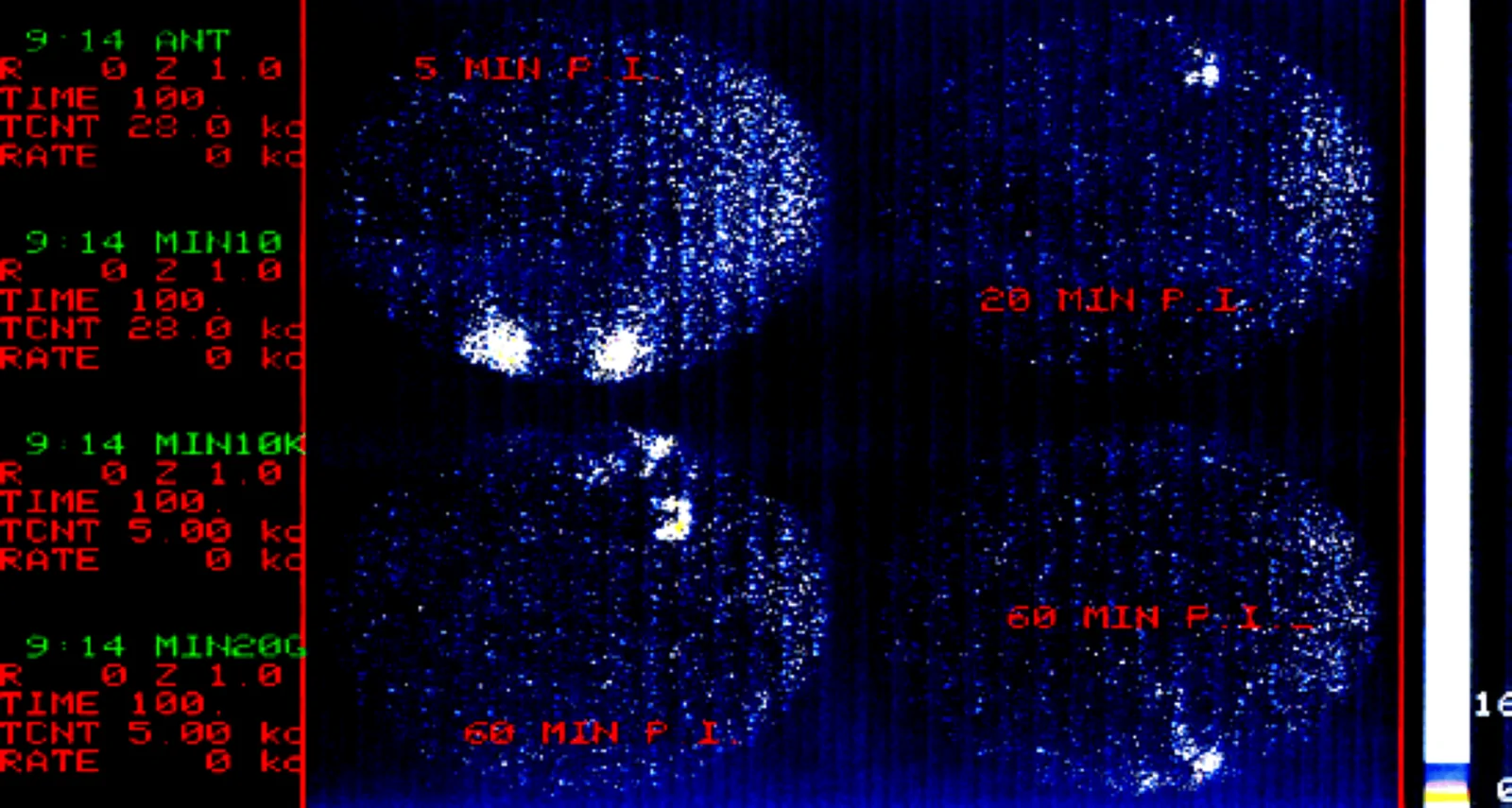
Lymphoscintigraphy performed 5, 20, and 60 min after isotope administration in the lower extremities.

## Discussion

Lymphedema is classified as primary or secondary. Primary lymphedema is further subdivided into congenital and hereditary forms [1]. Occasionally, congenital lymphedema may be associated with other congenital disorders. In this report, we describe a 15-year-old patient with a congenital cardiac anomaly, Ebstein anomaly, that was diagnosed during early infancy and who was subsequently found to have primary lymphedema. Thus, this patient presented with a combination of congenital abnormalities involving both the heart and the lymphatic system of the lower extremities.

Although the coexistence of Ebstein anomaly and primary lymphedema in our patient may be coincidental, we cannot exclude a possible developmental or genetic association. Both the cardiovascular and lymphatic systems develop during early embryogenesis, and abnormalities affecting mesodermal development may contribute to congenital defects involving both systems [3,4]. Moreover, several candidate genes have been identified as being involved in both cardiac and lymphatic vascular development, suggesting that shared genetic and developmental mechanisms may contribute to abnormalities affecting both systems [5]. Lymphatic abnormalities and dysfunction have also been described in patients with congenital heart disease, further supporting the biological plausibility of a developmental relationship between cardiac and lymphatic disorders [6]. Nevertheless, no specific association between Ebstein anomaly and primary lymphedema has been established, and further studies are required to determine whether their coexistence represents a true developmental association or is merely coincidental. In addition, air travel may have acted as a precipitating factor for the clinical manifestation of latent lymphatic dysfunction. Prolonged immobility and flight-related pressure changes have been associated with worsening edema and impaired lymphatic drainage [7]. In the present case, secondary causes of lower-extremity edema, including venous thrombosis, venous insufficiency, abdominal pathology, and cardiac deterioration, were excluded by appropriate imaging studies.

The findings in this case may suggest a previously unrecognized syndrome and warrant further investigation. Additional cases might be identified if patients with Ebstein anomaly undergo lymphoscintigraphic evaluation. This approach could facilitate early diagnosis of lymphedema and enable timely treatment, thereby reducing the physical and psychological burden on affected patients.

## Conclusions

In conclusion, this is a rare case of a 15-year-old patient with an Ebstein anomaly who was subsequently diagnosed with primary lymphedema of the lower extremities. This unusual coexistence may suggest a previously unrecognized association between congenital cardiac and lymphatic abnormalities. Although a causal relationship cannot be established based on a single case, the findings highlight the importance of considering lymphatic disorders in patients with congenital heart disease presenting with unexplained lower-extremity edema. Increased clinical awareness and further systematic evaluation, including lymphoscintigraphy when appropriate, may facilitate earlier diagnosis and improve patient management and outcomes.
